# The role of anxiety in patients with hereditary angioedema during oral treatment: a narrative review

**DOI:** 10.3389/froh.2023.1257703

**Published:** 2023-10-19

**Authors:** Alessio Rosa, Rocco Franco, Michele Miranda, Sergio Casella, Cesare D’Amico, Luca Fiorillo, Gabriele Cervino

**Affiliations:** ^1^ Materials for Health, Environment and Energy, Department of Chemical Science and Technologies, Dentistry, University of Tor Vergata, Rome, Italy; ^2^ Department of Life, Health and Environment Sciences, University of L’Aquila, L’Aquila, Italy; ^3^ Department of Clinical Sciences and Translational Medicine, University of Rome “Tor Vergata”, Rome, Italy; ^4^ Department of Biomedical and Dental Sciences, Morphological and Functional Images, University of Messina, Messina, Italy; ^5^ Department of Public Health Dentistry, Dr. D.Y. Patil Dental College and Hospital, Dr. D.Y. Patil Vidyapeeth, Pimpri, Pune, India; ^6^ Multidisciplinary Department of Medical-Surgical and Odontostomatological Specialties, University of Campania “Luigi Vanvitelli”, Naples, Italy

**Keywords:** angioedema, oral surgery, anxiety, C1 inhibitor, dental treatment

## Abstract

**Objective:**

The present study investigated the clinical potential of managing anxiety during dental procedures to reduce acute attacks in patients with hereditary angioedema (HAE). HAE is a rare disease, little known to physicians and dentists, but with an increased hospitalization rate over the years. HAE is due to a deficiency/dysfunction of the C1 esterase inhibitor, leading to increased vascular permeability. Recommendations for HAE management include long-term and short-term prophylaxis and treatment of acute attacks, but the importance of anxiety control is underestimated.

**Materials and methods:**

The authors reviewed the literature to provide the scientific community with an overview of possible protocols for managing anxiety in dental practice and their effectiveness. Management can be used in prosthetics, periodontal and implant surgery, endodontics, and oral surgery.

**Discussions:**

Our analysis shows that although there are few articles in the indexed literature, protocols for managing anxiety in HAE patients in dentistry will become increasingly prevalent in the daily clinical practice of dentists due to its benefits.

**Conclusions:**

The benefits and better control of intraoperative complications and risks may lead clinicians to use sedation, assessment, or anxiety control techniques in daily clinical practice to reduce such attacks. Clinical relevance: This study suggests that controlling and managing anxiety can help prevent and reduce acute angioedema attacks.

## 1. Introduction

Hereditary angioedema (HAE) is a rare genetic disease that affects 1:10,000–1:50,000 people. It has a chronic and disabling course and is difficult to diagnose without specialized evaluation. Diagnosis can be delayed up to 15 years after symptom onset (1). HAE is inherited as an autosomal dominant trait caused by a mutation in the C1-inhibitor gene on chromosome 11. This mutation results in a defect in C1 inhibitor, leading to uncontrolled activation of the complement system and the generation of vasoactive mediators that induce edema. There are two types of HAE: type I (85% of patients) characterized by a quantitative defect, and type II (15% of patients) characterized by a nonfunctioning protein. The most dangerous symptom is glottis edema, which can lead to asphyxiation (2). Treatment aims to prevent acute attacks and includes short-term prophylaxis before outpatient surgery and long-term treatment for patients with high attack rates. Experimental protocols and standardized guidelines are being developed for dental procedures. Psychophysical assessment and visual analog scales are useful for patient management. In 78% of patients with skin edema also shows facial edema and in most cases this phenomenon can affect the larynx, with change of voice (deep voice, hoarseness, aphonia), associated with dyspnea and feeling of suffocation. The purpose of the treatment is to avoid behaviours/therapies that favour the onset of attacks (3). The treatment is twofold: short-term prophylaxis to be carried out with Berinert or Cirnyze in the hours preceding any outpatient surgery; long-term treatment with the same drugs to be carried out in patients with high rates of acute attacks. Anxiety is one of the main causes of perioperative stress that affects the quality of life, increasing the perception of pain and compromising the result. In HAE cases, some factors including anxiety, emotional stress, trauma, infections, physical efforts, invasive medical procedures, and certain medications have been suggested as predisposing to HAE attacks leading to a vicious circle. However, further evidence is needed to establish a causal relationship between anxiety or emotional stress and the development of HAE attacks (4). Notwithstanding, managing anxiety has been suggested as a good strategy helping in the prevention of attacks and thus improving both patient prognosis and quality of care. The scarcity of literature on the management of this rare condition has necessitated the development of an experimental protocol for the drafting of standardized guidelines on dental procedures.

## 2. Materials and methods

This study followed the Preferred Reporting Items for Systematic Reviews and Meta-Analyses (PRISMA) statement. The main research question was captured in the Population, Intervention, Comparison, Outcomes (PICO) format, “Can anxiety management (I) in the preoperative phase (O) as reduce acute attacks (C) during dental care in patients with Hereditary Angioedema (P))? The search strategy involved searching electronic databases: the National Library of Medicine (Pubmed), Google Scholar, Scopus, Embase, Medline, and Cochrane Library databases were searched without time or language restriction to find articles describing the basic principles of systematic review and its applications in dental practice All studies reviewed were selected because they evaluate the clinical efficacy of anxiety management to prevent acute attacks in patients with hereditary angioedema. All studies reviewed were published between January 1, 2000, and April 30, 2023. The following word combination was used: “anxiety” AND “angioedema” AND “oral”; “anxiety” AND “angioedema” OR “oral”; “anxiety” AND “angioedema” OR “oral”; “anxiety” AND “angioedema” AND “oral”; “anxiety” AND “angioedema” AND “tooth”; “anxiety” AND “angioedema” OR “tooth”; “anxiety” AND “angioedema” AND “tooth”; “anxiety” AND “angioedema” AND “tooth”; “anxiety” AND “angioedema” AND “tooth”; “anxiety” AND “angioedema” AND “tooth”; “anxiety” AND “angioedema” AND “tooth”; “anxiety” AND “angioedema” OR “tooth”; “anxiety” AND “angioedema” AND “teeth”; “anxiety” AND “angioedema” OR “teeth”; “anxiety” OR “angioedema” AND “teeth”; “anxiety” OR “angioedema” OR “teeth”; “management” AND “angioedema” AND “child”; “management” AND “angioedema” OR “child”; “management” AND “angioedema” AND “child”; “management” AND “angioedema” AND “child”; “management” AND “angioedema” AND “oral”; “management” AND “angioedema” AND “dental”; “management” AND “angioedema” AND “dental”; “management” AND “angioedema” OR “dental”; We included all available levels of evidence (including case reports, *in vitro* studies, animal model studies, and case series). No comments or letters to the editor were requested.

### 2.1. Synthesis of results

**Figure 1:**
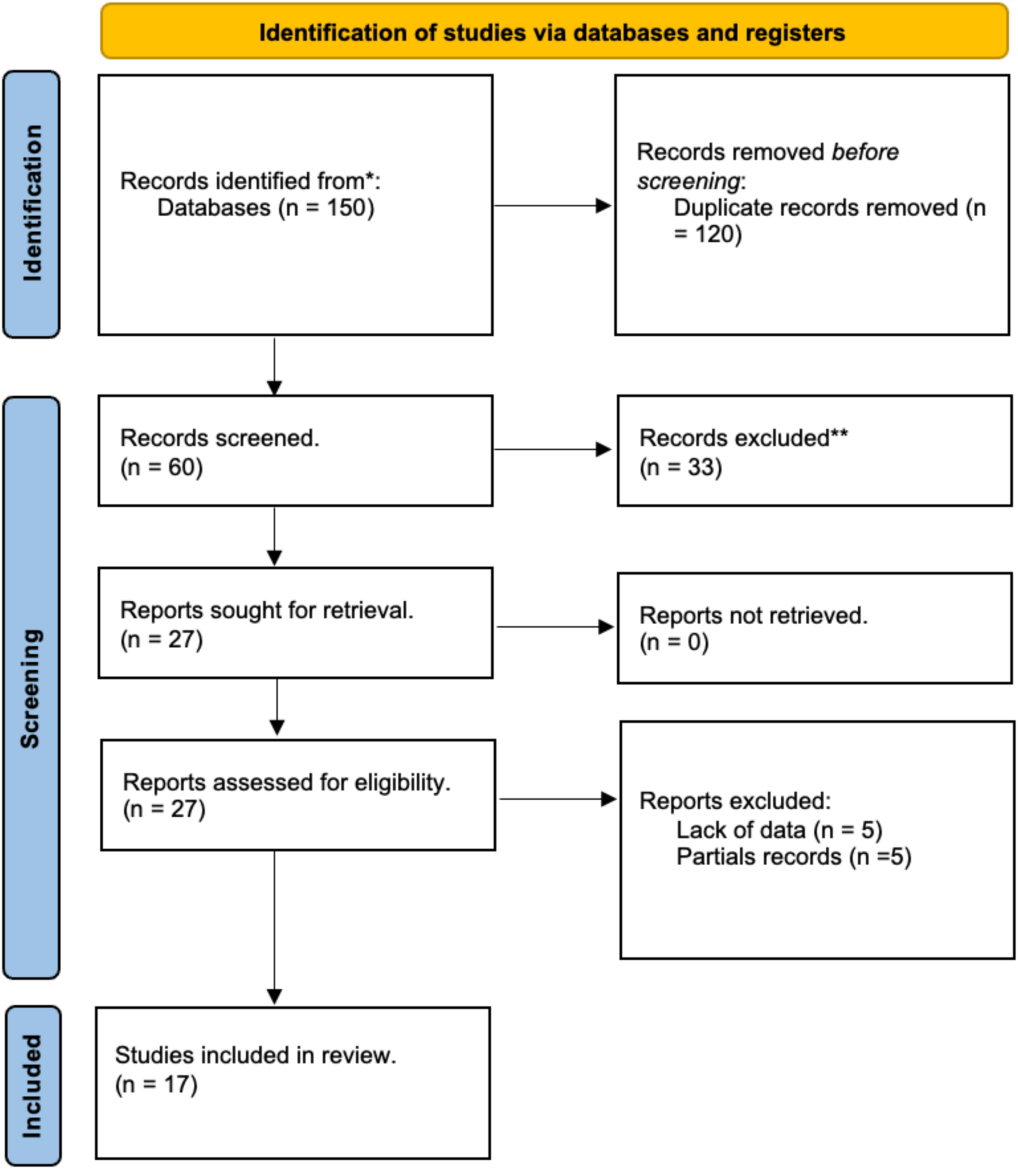
Search strategy flow chart.

At the conclusion of the search, 150 studies were identified through the five databases. 60 articles were included in the initial screening phase, 33 articles after initial screening were removed. Abstracts of 17 publications were evaluated during the final screening process and included in this review (Figure 1).

## 3. Discussion

### 3.1. Anxiety and oral surgery distress

Anxiety disorders are highly prevalent among patients with chronic illnesses and are frequently observed in individuals with HAE. The uncertainty and unpredictability of HAE attacks, the fear of asphyxiation during a laryngeal attack, and the potential impact on social and professional life can all contribute to anxiety. Anxiety can significantly impact disease management in patients with HAE (3, 5, 6). Anxiety-induced stress can potentially worsen an acute HAE attack. The international WAO/EAACI guidelines 2002 recognize that surgical trauma, dental procedures, and other procedures associated with mechanical impact on the upper aerodigestive tract (e.g., endotracheal intubation, bronchoscopy, or esophagogastroduodenoscopy) can precipitate angioedema near the surgical site. After tooth extraction, more than one third of patients without preprocedural prophylaxis may develop local angioedema, so preprocedural prophylaxis is extremely important because it reduces the risk of angioedema associated with these procedures. Short-term prophylactic treatment before medical, surgical, or dental procedures and before exposure to other angioedema-inducing events is recommended (7). Moreover, anxiety may interfere with treatment adherence and regular follow-up, affecting overall disease control. Anxiety is a major cause of perioperative stress as it increases pain perception and can alter and compromise surgical outcomes. The VAS is a valid test for preoperative anxiety, with greater sensitivity than other scales. It can also be used alone to improve the assessment of dental anxiety. Patients with a VAS-A >5.0 cm should be considered anxious and those with a VAS-A ≥7.0 as phobic. With these scales, there may be greater sensitivity in anxiety control and management of acute attacks (8). In another study, Van Sickels showed that the mean diadidic adjustment scale (DAS) score in a population is higher in dental clinic patients than those reported in community studies (9). Also Zanette in another study (2) showed how patients with systemic diseases have more anxiety than patients who are not systemically compromised. These have more risk of developing an acute attack during the perioperative phase because they already have preoperative anxiety (10–12).

Because HAE is a sporadic and still unknown condition, hospitalization rates have increased in recent years. Diagnosis and proper treatment may be delayed for years because, still clinically the signs and symptoms are doubtful. However, this means that patients may receive ineffective or inappropriate treatment, sometimes subjecting themselves to unnecessary medical care resulting in physical and psychological disabilities that can generate high levels of anxiety, depression and poor quality of life (13). The first step in managing a patient with Angioedema is to perform a proper psychophysical assessment. In a very interesting study by Alkanan et al. there is evidence to support the use of aromatherapy to better manage preoperative anxiety in adults before undergoing dental care. Following this study, lavender oil, citrus preparations, and rose oil were shown to be among the aromatherapy compounds with the greatest efficacy in reducing anxiety. Inhalation of these compounds at short duration (≤20 min per session) appears to be practical and feasible. However, it is obvious to seek further randomized controlled trials to generate high-quality scientific evidence, clarify the basis of the mechanisms of action of aromatherapy, and then develop new optimal aromatherapy protocols to improve preoperative anxiety (14). In this regard, it may be interesting to apply this therapeutic field in the preventive management of the acute attack of HAE. Although HAE is rare, its relevance in dentistry remains essential because of its life-threatening consequences. Several deaths following extractions in both adults and children with HAE have been reported by Forrest et al. in the literature, most notably a delayed onset of laryngeal asphyxia up to two days after surgery (15). This highlights the need for strategic planning using a multidisciplinary approach that follows the guidelines in the literature. Cinquini et al. report that facial swelling and laryngeal edema appeared in 21.5 percent of dental extractions without C1-INH concentrate prophylaxis compared to 12.5 percent of cases in those who received C1-INH prophylaxis (13). This should therefore support the decision to administer prophylactic C1 esterase inhibitor concentrate preoperatively in HAE patients. Therefore, hospital monitoring in such patients should be strongly considered ideally with the level of care. It is known that anxiety triggers HAE, so patients' psychological and holistic well-being should be put on the same level as their physical health during the preparatory phase for any surgery or treatment. The case has highlighted the vital importance of communication and multidisciplinary work during the planning, pre-, peri- and post-operative aspects of care in complex issues such as this one. HAE patients represent a special category as they require careful pre/intra/post-operative procedures. Therefore, a correct and accurate knowledge of the pathology is indispensable both from a clinical and pathological point of view so that any difficulties can be managed. The initial support of the allergist/immunologist is fundamental, subsequently the patient must be informed in detail about the risks and possible complications of the procedure (16). It is important that the operator knows that emotional stress is today one of the main triggers of angioedema attacks and precisely for this reason conscious sedation techniques during oral and maxillum surgery. Facial are the first choice to prevent acute attacks (17). Even minor procedures such as pulpectomy or simply detecting a mobile prosthesis impression can be responsible for the onset of an attack. HAE patients should not undergo any procedure without carrying out a precise preparatory prophylaxis. The patient's opinion on the choice of procedures to be carried out is also important, since the most invasive ones should be avoided as much as possible or in any case accepted by the patient once his consent has been expressed. Even only intubation itself can trigger a laryngeal attack, many authors, in fact, demonstrate how sedation is preferred in literature rather than general anesthesia (18).

Moraes et al. have enucleated a dentigera cyst in an 8-year-old pediatric patient with HAE under general anesthesia after prophylaxis with the drug danazol. Due to a recurrence of the cyst nine months after the first surgery and prophylaxis was administered in the form of fresh frozen plasma, however, both interventions succeeded without complications dangerous for the life of the small patient (19).

### 3.2. Anxiety and paediatric population in dentistry

Dental fear and dental anxiety are often used interchangeably in the scientific literature, but they represent different progressive degrees of the same psychological condition. The terms dental fear and dental anxiety (FA) analyzed in this study are related to dental treatment among children and adolescents. This abnormal childhood or adolescent dental anxiety can sometimes be related to various uncooperative or annoying behaviours. This fear associated with the dentist, with varying degrees of severity, is a phenomenon described in studies conducted in different geographic areas, such as Western Europe as well as poor oral health in children (8).

Eijlers in a review showed that the prevalence of fear and anxiety (FA) in pediatric populations was significant in different settings (20). At least one in ten children had a level of FA that hindered their ability to tolerate dental care. These data are similar to those reported by Klingberg et al. in a previous systematic review (21). In studies using the VAS scale, younger boys and girls had higher FA values. Children and adolescents in northern Europe had lower prevalence and lower levels of AF than their peers in other geographic areas. Other population variables, such as type of questionnaire respondent (children or parents/guardians proxy) and environment (school or dental clinic) were not significantly related to AF. Each study's choice of cut-off level also influenced the prevalence of FA (21).

It is therefore extremely important to intercept dental fear, underline the identification of the triggering factors and develop prevention to reduce its onset. Moraes examined preoperative and postoperative anxiety in children with HAE in a study and found that children with HAE show higher preoperative anxiety than controls of healthy children (19).

One might idealize that children with HAE bear the brunt of the disease burden in the same way as adult patients (22).

Rosa has shown that physical and psychological stress can lead to a worsening clinical picture in HAE patients during routine dental procedures; it is extremely important to assess anxiety in children as well, as they are unable to self-assess their anxiety state (3). Zotter also hypothesized that at the top of the etiological pyramid of the most common triggers of HAE present was emotional distress due to preoperative anxiety (23).

With this mini-review it has been shown that the number of attacks is a significant factor in differentiating the anxiety level of children with HAE compared to the anxiety level of children without episodes and healthy control children. However, the school phase does not allow to accurately determine whether HAE disease activity increases the anxious trait because pediatric patient compliance is reduced. It is essential to produce intervention programs that focus on the physical and emotional aspects of quality of life. life of children with HAE. This approach could reduce the chances of triggering a bout of illness, increase patients' sense of control and independence, and improve the chances of long-term success in their careers and personal lives. It may also reduce the transition from high anxiety trait to anxiety disorder (24). Moraes demonstrated that oral management of a pediatric HAE patient can be successfully performed if the health care providers know the risks and prevention strategies. In this work, enucleation of a dentigerous cyst on an 8-year-old patient was performed by preemptively limiting the acute attack through medication. Thus, success depends on a multidisciplinary professional relationship and a careful treatment plan. However, health care providers must be prepared for unwanted angioedema episodes such as acute attacks (19) (Table 1).

**Table 1:** Features of HAE patients of included studies.

Age-gender-diagnosis	Dental treatment	Type of prophylaxis	Previous attacks	Measuring scale
17 - F - HAE	3 EX	C1 INH	2–3 Y	VAS
32 - F - HAE	3 EX	C1 INH	1 Y	CDAS - VAS
74 - F - HAE	1 EX + 1 RC	C1 INH	NOT REPORTED	CDAS
42 - F - HAE	3 EX	C1 INH	4 Y	VAS
29 - M - HAE	2 EX + 1 CT	C1 INH	1 Y	CDAS - VAS
34 -M -HAE	3 DI	C1 INH	1 Y	CDAS

### 3.3. Anxiety and pharmacologic sedatives

Accumulating evidence has revealed that dental anxiety, as a dispositional factor toward the dental situation, is associated with state anxiety and pain related to dental procedures. However, the conclusions of individual studies may be limited by the treatment procedures received by patients, the instruments used to assess anxiety, or the stages of treatment at which anxiety or pain were evaluated (22). The impact of this on pain at different stages of treatment has been systematically studied. In the specific case of angioedema, anxiety has been suggested as one of the factors predisposing to acute attack. Lin in a review with meta-analysis, revealed that studies on surgical and non-surgical procedures did not differ significantly for either dental anxiety or pre-treatment anxiety. It is equally essential to assess preoperative anxiety as a critical phase for all highly odontophobic patients, which is also critical in pain control for all dental patients (25). Pharmacological treatment is indispensable to make this critical phase manageable and includes inhalation agents such as nitrous oxide, orally or parenterally administered drugs such as midazolam and other sedative-hypnotic or psychostimulant. It has been confirmed in the literature that when used in combination these drugs produce a synergistic effect and parents themselves more commonly accept sedation with laughing gas rather than midazolam (25). The present mini review identified the threshold level of sedation produced by a combination of nitrous oxide and parenteral midazolam in adults and children in order to achieve careful anxiety management. Sivaramakrishnan, in a meta-analytic systematic review, identified the use of the combination of nitrous oxide and midazolam as the primary sedation technique for dental treatment in adults and children compared to the individual use of nitrous oxide or midazolam, respectively (26). This gas is generally administered in varying concentrations ranging between 30 and 40 percent with oxygen through a very practical and ergonomic nasal mask. Humphris demonstrated the use of midazolam as a premedication for sedation and induction of general anesthesia in a randomized clinical trial. It is commonly administered intramuscularly or intravenously. The most common different routes of administration are the oral and the nasal one, obviously they are preferred in children precisely to avoid the anxiety of the needle and therefore potential preoperative stress, which is counted as the most common and subtle cause of acute attack (27).

The manuscript discusses the clinical potential of managing anxiety during dental procedures to reduce acute attacks in patients with hereditary angioedema HAE. HAE is a rare genetic disease characterized by a deficiency or dysfunction of C1 esterase inhibitor, leading to increased vascular permeability. Using visual-analog scales VAS to assess perioperative anxiety and the implementation of conscious sedation are discussed as effective strategies (27). Additionally, the study explores the potential benefits of aromatherapy in improving preoperative anxiety. The rarity and lack of awareness about HAE among physicians and dentists contribute to delayed diagnosis and inappropriate treatment. This further emphasizes the need for proper psychophysical assessment and management of patients with HAE. The discussion underscores the importance of multidisciplinary collaboration and communication in planning, pre-, peri-, and post-operative care for HAE patients (28, 29).

Therefore, the first essential step in correctly managing the patient is to conduct a psycho-physical evaluation of the subject (4).

## 4. Conclusions

The present study emphasizes the need for a multidisciplinary and multi-component approach, incorporating proper psychophysical assessment and anxiety control techniques, in order to improve the management of patients living with angioedema. The results of this study have clinical relevance and provide a basis for further research in this field. Further research in this regard will be needed to expand and strengthen the basic concept that anxiety fits into the etiology of acute HAE attacks.
